# Ex Vivo Evaluation of Mechanical Anisotropic Tissues with High-Frequency Ultrasound Shear Wave Elastography

**DOI:** 10.3390/s22030978

**Published:** 2022-01-27

**Authors:** Seungyeop Lee, Lucy Youngmin Eun, Jae Youn Hwang, Yongsoon Eun

**Affiliations:** 1 Department of Information and Communication Engineering, Daegu Gyeongbuk Institute of Science and Techonology (DGIST), Daegu 42988, Korea; lsy1218@dgist.ac.kr (S.L.); jyhwang@dgist.ac.kr (J.Y.H.); 2 Division of Pediatric Cardiology, Department of Pediatrics, Yonsei University College of Medicine, Seoul 06229, Korea

**Keywords:** elastography, mechanical anisotropy, high-frequency ultrasound, mechanical wave imaging, heart experiment, cartilage experiment

## Abstract

The use of imaging devices to assess directional mechanics of tissues is highly desirable. This is because the directional mechanics depend on fiber orientation, and altered directional mechanics are closely related to the pathological status of tissues. However, measuring directional mechanics in tissues with high-stiffness is challenging due to the difficulty of generating localized displacement in these tissues using acoustic radiation force, a general method for generating displacement in ultrasound-based elastography. In addition, common ultrasound probes do not provide rotational function, which makes the measurement of directional mechanics inaccurate and unreliable. Therefore, we developed a high-frequency ultrasound mechanical wave elastography system that can accommodate a wide range of tissue stiffness and is also equipped with a motorized rotation stage for precise imaging of directional mechanics. A mechanical shaker was applied to the elastography system to measure tissues with high-stiffness. Phantom and ex vivo experiments were performed. In the phantom experiments, the lateral and axial resolution of the system were determined to be 144 μm and 168 μm, respectively. In the ex vivo experiments, we used swine heart and cartilage, both of which are considered stiff. The elastography system allows us to acquire the directional mechanics with high angular resolution in the heart and cartilage. The results demonstrate that the developed elastography system is capable of imaging a wide range of tissues and has high angular resolution. Therefore, this system might be useful for the diagnostics of mechanically anisotropic tissues via ex vivo tests.

## 1. Introduction

The mechanical properties of biological tissues, including muscle, skin, tendons, cartilage, fat, etc., are closely related to pathological states as diseases alter the mechanical properties of tissue. Based on this relation, a clinical examination method called palpation, which qualitatively assesses mechanical properties of tissue with the fingers or hands, has been used for thousands of years to diagnose diseases. Until several decades ago, palpation was only possible on the surface or shallow depths of the body. Since the introduction of elastography in the 1990s, remote palpation inside the human body has also become possible [1].

Several remote palpation methods based on magnetic resonance imaging and ultrasound imaging have been developed. Among these elastography methods, ultrasound-based elastography has important advantages such as higher temporal resolution, low patient exclusion rate, low cost, portability, frequent use by elastography technicians, and widespread availability in hospitals and clinics worldwide [2]. Thus, ultrasound-based elastography is widely used in the diagnostics of many diseases such as cancer, fibrosis, etc.

Some tissues require high spatial resolution of hundreds of micrometers for proving high quality image for disease diagnostics. Specifically, a heart consists of several layers such as endocardium, myocardium, epicardium, etc. In particular, each layer has the thickness of 0.5∼5 mm. The quantification of directional mechanics of each layer would be beneficial for the better diagnostics of the heart diseases. In addition, high-frequency ultrasound imaging showed the usefulness for eleastography of tissues with thickness less than few millimeters in the previous study [3]. Thus, high-frequency ultrasound elastrography is required.

The most commonly used ultrasound-based elastography measures mechanical properties by generating localized displacement in tissue and detecting the movement caused by the displacement [4,5,6]. It primarily uses acoustic radiation force (ARF) to generate localized displacement [7]. However, for hard tissues such as the heart, it is difficult to generate sufficiently localized displacement with ARF: for enough displacement, ARF excitation must deliver high-energy pulses, which may easily damage high-frequency ultrasound elements. In addition, the resulting high negative pressure exceeds the standards of the US Food and Drug Administration for in vivo applications [8,9].

The stiffness of human tissues varies. For example, the brain tissue is soft whereas the tendon, ligament, and cartilage are hard [10,11]. Diseases change the mechanical properties of all of these tissues. Therefore, for the diagnostics of various diseases, mechanical properties of tissues with both low-stiffness and high-stiffness need to be measured. However, elastography systems for high-stiffness tissues with elastic moduli greater than tens of kilopascals, such as cartilage, have not been explored due to the difficulty of generating enough displacement in high-stiffness tissues with ARF. To overcome this problem, a shear wave elastographic systems using a mechanical shaker as a shear wave source for the high-stiffness tissue experiments were developed in the previous studies [12,13]. In this study, we combined the mechanical shaker with a high-frequency ultrasound transducer to develop a high resolution mechanical wave imaging system that can be used in the measurement of mechanics of tissues with high-stiffness.

Most previous studies assume that the mechanical properties of tissue are homogeneous and isotropic, especially for applications in the liver, breast, and prostate [14,15,16,17]. On the other hand, many other tissues are mechanically anisotropic, which means that the mechanical properties of the tissues vary with measurement direction [18,19]. Ignoring the measurement direction will make the diagnostics inaccurate and unreliable. Commercial probes are limited in their ability to measure directional mechanics. As commercial probes do not provide a rotation function, the probe or tissue must be manually rotated to measure directional mechanics. However, such manual rotation is not only time consuming but also reduces the accuracy in the measurement of directional mechanics of tissues of interest. A motorized rotation stage with high enough angular resolution (minimum angle that the stage rotates) is desired for the measurement of direction-dependent mechanical properties. Although some groups have studied mechanical anisotropy with elastography, these imaging systems require manual rotation [19] or provide insufficient angular resolution [20,21,22].

In this paper, we developed a high-frequency mechanical wave elastography system to measure directional mechanics in a wide range of tissues regardless of stiffness. A motorized rotational stage is applied to the elastography system to detect directional mechanics. This motorized rotational stage allows the system to have high angular resolution, thus increasing the accuracy and reliability of diagnostics. In addition, the system stimulates tissues with a mechanical shaker to generate enough displacement in high-stiffness tissues. This approach eliminates the need for the ultrasound transducer to generate ARF. Thus, the transducer frequency can be sufficiently high to satisfy the image resolution requirements. In other words, this system can acquire high-resolution images and high-precision mechanical properties depending on the measurement direction.

Phantom experiments were performed to assess the performance of the system in terms of lateral and axial resolution and showed how small a deformation the system can measure.

To demonstrate the imaging capability for stiff anisotropic tissues, we selected swine heart and cartilage. The heart is considered a stiff material, with a shear modulus in the range of 5∼25 kPa depending on diastole/systole, and cartilage is much stiffer, with a shear modulus on the order of MPa. The swine heart experiments indeed show that wave speeds differ for different measurement directions indicating that the mechanical anisotropy is important factor to be examined. The cartilage experiments are performed to show that the system is also useful for measuring directional mechanics of tissues with stiffness in the order of MPa.

## 2. Materials and Methods

### 2.1. Imaging Hardware Setup

**Figure 1:**
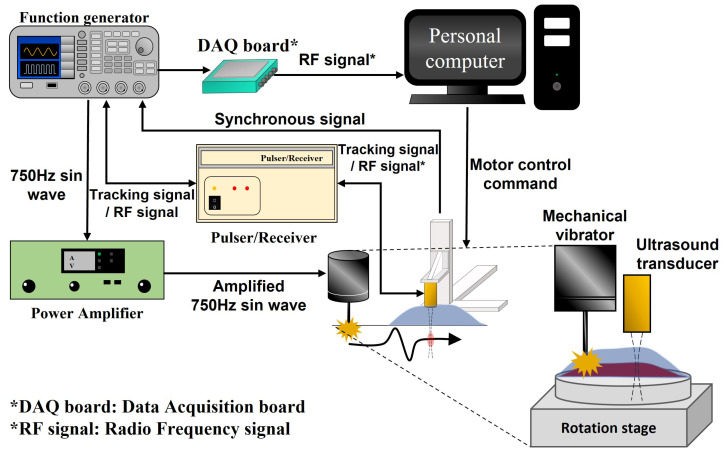
Experimental setup for the mechanical wave imaging system.

The mechanical wave imaging system works by generating mechanical waves in tissue and subsequent motion tracking of the induced tissue motion caused by the mechanical waves. Figure 1 presents the experimental setup of our acquisition system for the implementation of high-frequency ultrasound mechanical wave imaging. A detailed description of system operation is given in the following paragraphs.

The user controls the motorized stage (OSMS26-100, Sigma Koki, Tokyo, Japan) through a personal computer, and the motorized stage sends trigger signals to the function generator (AFG 3052C, Tektronix, Beaverton, OR, USA). Then, the function generator synchronously triggers the mechanical shaker (mini-shaker type 4810, Bruel & Kjaer, Duluth, GA, USA), the ultrasound transducer, and the personal computer to acquire the tissue motion data. The shaker is operated with an excitation signal generated by a function generator and amplified by a power amplifier (amplifier type 2718, Bruel & Kjaer, Duluth, GA, USA). The shaker generates a tone burst of 750 Hz on the tissue surface, and a mechanical wave is propagated from the excitation point to the observation area. A 38 MHz single-element high-frequency ultrasound transducer is operated with a tracking signal generated by the function generator and a pulser/receiver (DPR500, JSR Ultrasonics, Pittsford, NY, USA). Then, the ultrasound transducer generates acoustic waves to track and record the induced tissue motion. The target tissue is attached to a motorized rotation stage, which rotates the target tissue to analyze the directional mechanics of the tissue. The ultrasound transducer is attached on a 3-axis motorized stage, which transports the transducer to the imaging area. In the system, all motorized stages are controlled by a computer via a step motor controller (SHOT-304GS, Sigma Koki, Japan). The transducer moves while the imaging system operates, and all of the above processes are repeated at each imaging location.

**Figure 2:**
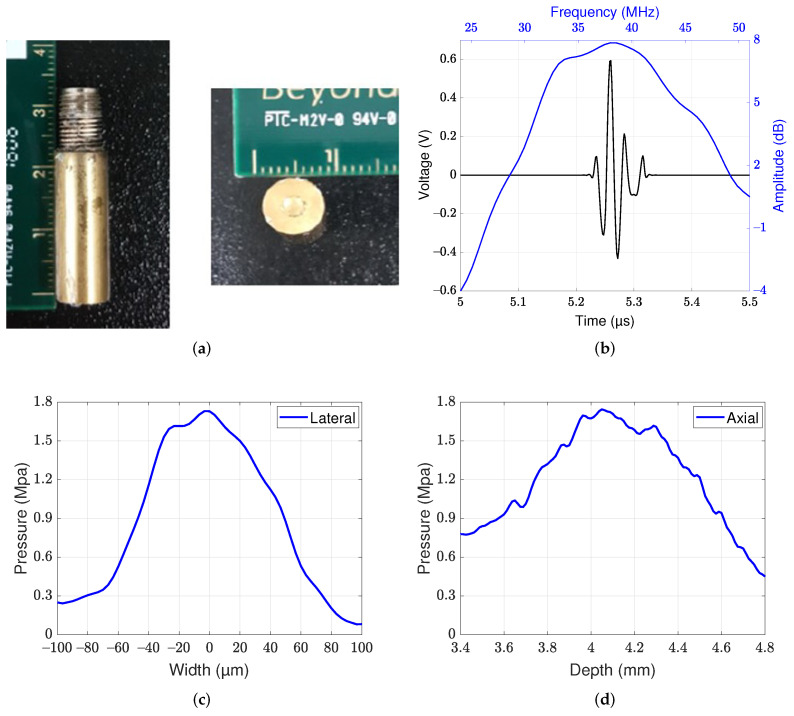
Characteristics of the 38 MHz lithium niobate ultrasound transducer at the focus of ultrasound beam. (**a**) Photographic image of the transducer, (**b**) pulse-echo characteristics of the transducer, (**c**) lateral beam profile, and (**d**) axial beam profile.

A 38 MHz lithium niobate ultrasound transducer was fabricated and attached to the 3-axis motorized stage to allow tracking of the displacement caused by mechanical waves. A photographic image of a single-element lithium niobate ultrasound transducer is shown in Figure 2a. The center frequency of the transducer was 38 MHz, and the −6-dB bandwidth was 47.5% (Figure 2b). The aperture diameter and focal lengths were 2.5 mm and 4.05 mm, respectively, and the f-number was 1.62. The −6-dB lateral and axial beamwidths were measured to be 90 μm and 1.1 mm, respectively, by quantification of the beam profiles at the focus level (Figure 2c,d). In the acoustic intensity measurement system, a hydrophone (HGL-0085, ONDA) was used to measure the beam profiles. The diameter of the hydrophone was 85 μm.

### 2.2. Procedure for Mechanical Wave Imaging

A wave speed is calculated from data acquisition and multiple signal processing. The details are as follows:

#### 2.2.1. Data Acquisition

Data acquisition is initiated by moving the motorized stage. The motorized stage moves at a speed of 250 μm/s, while the step motor controller generates trigger signals transmitted to the function generator every 50 μm. The function generator generates a 1-cycle 750 Hz tone-burst sinusoidal signal for the mechanical shaker and 500 trigger signals that have a pulse repetition frequency of 20 kHz for the ultrasound transducer. In this system, the frequency of the mechanical wave was determined to be 750 Hz, because reference [8] shows that mechanical wave measurement was stable above 300 Hz, and our own experiment (presented in Section 3.1) support this observation as well.

Every 50 μm, the mechanical shaker stimulates the tissue surface with a single sine wave of 750 Hz, while the ultrasound transducer transmits a focused ultrasound beam and acquires the backscattered radio frequency signal. Transmission and acquisition repeat for 25 ms. This procedure is repeated up to a depth of 2 mm at 500 μm intervals. All of the above procedures are repeated every 3${}^{\circ }$ (step angle interval) to measure the directional mechanics.

#### 2.2.2. RF Signal Filtering

The backscattered radio frequency signals are interpolated in the time domain by cubic spline interpolation. The interpolated signals are filtered with a bandpass filter with cutoff frequencies of 36 MHz and 40 MHz to reduce low- and high-frequency noise. After filtering, the filtered signals are used to estimate tissue displacement.

#### 2.2.3. Displacement Estimation

The displacement of tissue is estimated by applying the conventional cross-correlation method to the bandpass filtered signals [23]. The cross-correlation window length is four times the ultrasound wavelength. After estimating the displacements, the estimated displacement data are filtered with a bandpass filter to suppress low- and high-frequency noise. The bandpass filter with cutoff frequencies of 650 Hz and 850 Hz is used to extract the 750 Hz sine wave. The extracted displacement maps with a frequency of 750 Hz are used to estimate the wave speed.

#### 2.2.4. Wave Speed Estimation

The extracted displacement maps include not only forward waves but also backward waves that occur from reflection. The reflected backward waves make wave speed estimation difficult; thus, the backward waves must be removed with a directional filter as described in reference [24]. The directional filter mask removes the backward waves, and the filtered waves are used to estimate the wave speed.

The wave speed in the tissue is estimated from the slope of the time delay in the displacement maps (moving distance vs. estimation time). The time delay is calculated from the cross-correlation between the wave at the start point and the wave at each point. The slope of the time delay is linearized with linear regression, and the linearized slope represents the wave speed. Wave speed map consists of estimated wave speed at each point. Each wave speed map is filtered with 5 × 5 median filter to acquire a smooth image.

### 2.3. Phantom Experiments

Phantom experiments were designed to validate the developed imaging system and to identify the spatial resolution of the imaging system. Heterogeneous phantom experiments were designed for the above purposes. The heterogeneous phantoms were divided into layered phantoms for the axial performance and side-by-side phantoms for the lateral performance. Each heterogeneous phantom is made with two concentrations to divide the mechanical property laterally and axially. All phantom experiments were repeated three times for each phantom for more accurate measurements.

### 2.4. Phantom Preparation

Two types of tissue-mimicking phantoms, namely, a layered phantom and side-by-side phantom, were fabricated with agar powder. These phantoms were primarily constructed to identify the spatial resolutions. The step-by-step procedure for phantom fabrication was as follows [25]. First, the agar powder was presoaked in deionized water and then heated and stirred until it reached the gel point ($80{\;}^{\circ }C$). After the whole solution became transparent, n-propanol was added to the solution to increase the sound speed, and graphite powder was added to provide the ultrasonic backscattering signal. The solution was then cooled to $30{\;}^{\circ }C$ (near its congealing point) in an ice water bath, and a small quantity of paraformaldehyde was added to increase cross-linking. Finally, the solution was poured into a container and stored in a refrigerator. The above procedure was for a homogeneous phantom at a particular concentration. Each heterogeneous phantom is made from two homogeneous phantoms with different concentrations (1 wt% and 1.50 wt%). The layered phantom was made by pouring 1 wt% agar solution on the homogeneous phantom with 1.5 wt% concentration, and the side-by-side phantom was made by pouring 1 wt% agar solution at the side of the homogeneous phantom with 1.5 wt% concentration.

### 2.5. Animal Experiments

Swine hearts and cartilage were purchased from an online butcher shop that provides meat within one day after butchering. After purchase, the samples were stored in a refrigerator at $3{\;}^{\circ }C$ and measured within one hour after being removed from the refrigerator. Each heart was dissected to obtain the left ventricle. The left ventricle was measured to observe the directional mechanics of the heart. The left ventricle was also measured at each direction, each location, and each layer to assess the variation of mechanical anisotropy in each area. In addition to heart experiments, cartilage experiments were performed to determine whether the developed system is capable of analyzing the mechanical anisotropy of high-stiffness tissue. Both heart and cartilage experiments were performed with high angle resolution to obtain the directional mechanics. In the experiments, the left ventricles and cartilages are cut into cuboid shapes (2 cm, 2 cm, 1 cm for the heart, 2 cm, 2 cm, 2 mm for the cartilage) so that the measurement system has access to various directions.

### 2.6. Calculation of Spatial Resolution

The spatial resolutions of the imaging systems were determined through R${}_{2080}$ methods [26]. The resolutions were calculated by fitting the obtained wave speed profiles to a sigmoid function. The sigmoid function was used to model the different speed values of the phantom. The function is described as follows:

(1) $$V_{s}\left(x\right)=\left(V_{1}-V_{2}\right)\left(\frac{1}{1+e^{\frac{-(x-x_{1})}{\lambda }}}\right)+V_{2},$$

where *x* is the axial or lateral distance in the phantom and $V_{1}$ and $V_{2}$ are the measured wave speed in the phantom with different concentrations, respectively. $x_{1}$ is the location of the layer boundary, and λ represents the transition width between two layers. From the wave speed profile, the four parameters $V_{1},V_{2},x_{1},$ and λ can be obtained with standard nonlinear least-squares fitting procedures. The spatial resolution is defined as a width between 20% and 80% transitions of the wave speed profiles and is calculated as follows:

(2) $$R_{2080}=\frac{\sum_{i=1}^{N}2ln\left(4\right)\left|\lambda_{i}\right|}{N},$$

where *N* is the total number of wave speed profiles across two layers in each phantom (the side-by-side and layered phantoms). The parameter $\lambda_{i}$ is the width of the *i*th transition of the wave speed profile. The mean resolution of our imaging system was obtained by averaging the transition widths estimated in different lateral or axial positions.

## 3. Results

### 3.1. Identification of the Spatial Resolution

**Figure 3:**
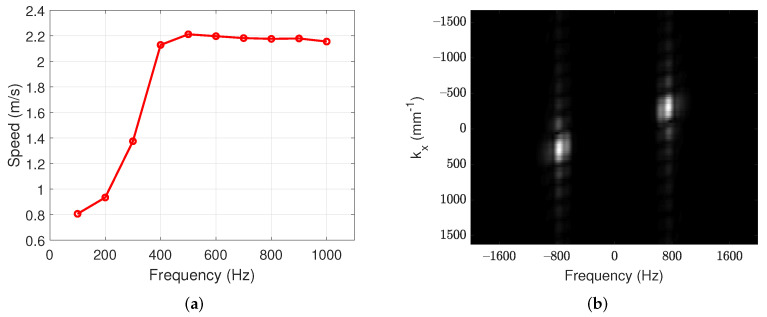
The results of mechanical wave elastography in homogeneous phantom. (**a**) Wave speed for various excitation frequencies and (**b**) frequency spectrum on excitation frequency of 750 Hz.

Figure 3a shows wave speed for various excitation frequencies. The speeds at low frequencies are slower, which is known to be caused by the viscosity and thickness of the material [27]. Figure 3a indicates that the excitation frequency should be greater than approximately 400 Hz for stable measurement. This result is similar to the result of [8]. For the proposed system, the excitation frequency of 750 Hz was chosen. One can see in Figure 3a that, at around 750 Hz, the speed measurement is insensitive to frequency variation. Furthermore, Figure 3b represents frequency spectrum on the homogeneous phantom with excitation frequency of 750 Hz. This figure shows that most of frequency components are located at 750 Hz.

**Figure 4:**
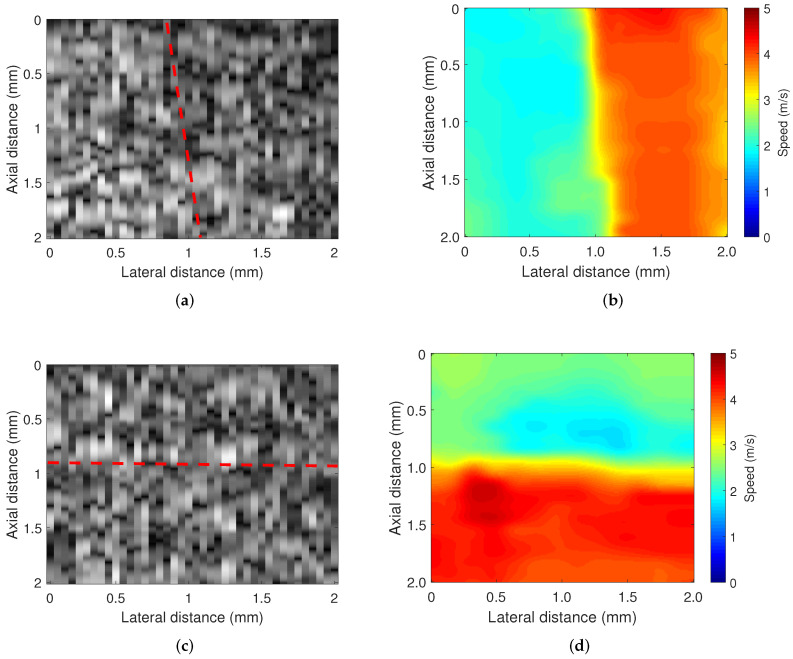
B-mode image and its corresponding wave speed image for heterogeneous phantoms. The red dotted line indicates the boundary between two phantoms. (**a**) B-mode image: side by side phantom, (**b**) elastography: side by side phantom, (**c**) B-mode image: layered phantom, and (**d**) elastography: layered phantom.

The spatial resolutions of the imaging system were determined in two types of heterogeneous phantom experiments. Figure 4 presents the B-mode images and wave speed images. The side-by-side phantom was made with 1.0 wt% and 1.5 wt% agar concentrations for left and right, respectively. The layered phantom was made with 1.0 wt% and 1.5 wt% agar concentrations for the top and bottom, respectively. In this figure, the heterogeneous structures of the agar phantom are not visible in the B-mode images. However, these heterogeneous structures are clearly visible in the wave speed images. The estimated wave speed in each phantom was 1.9 m/s and 3.9 m/s for the side-by-side phantom and 2 m/s and 4.2 m/s for the layered phantom at the agar concentrations of 1.0 wt% and 1.5 wt%, respectively.

**Figure 5:**
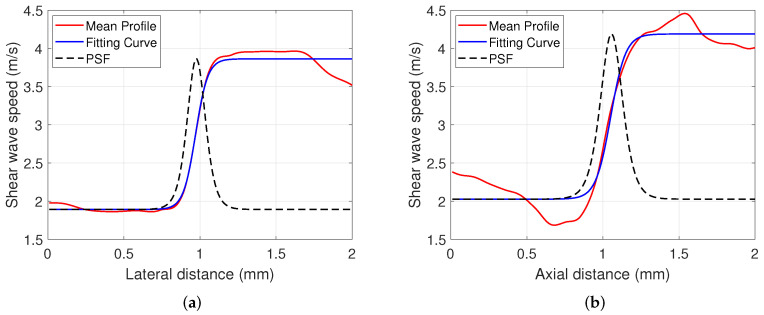
Axial and lateral resolution determination for mechanical wave elastography. (**a**) Lateral direction and (**b**) axial direction.

The lateral and axial resolutions were calculated to be 144 μm and 168 μm, respectively. Figure 5 presents the average wave speed profile and fitting curve in each phantom. Furthermore, the angular resolution is 3 degrees, which is the minimum angle at which the stage rotates the tissue in the imaging process.

### 3.2. Animal Experiments: Measurement of the Directional Mechanics

#### 3.2.1. Heart Experiments

**Figure 6:**
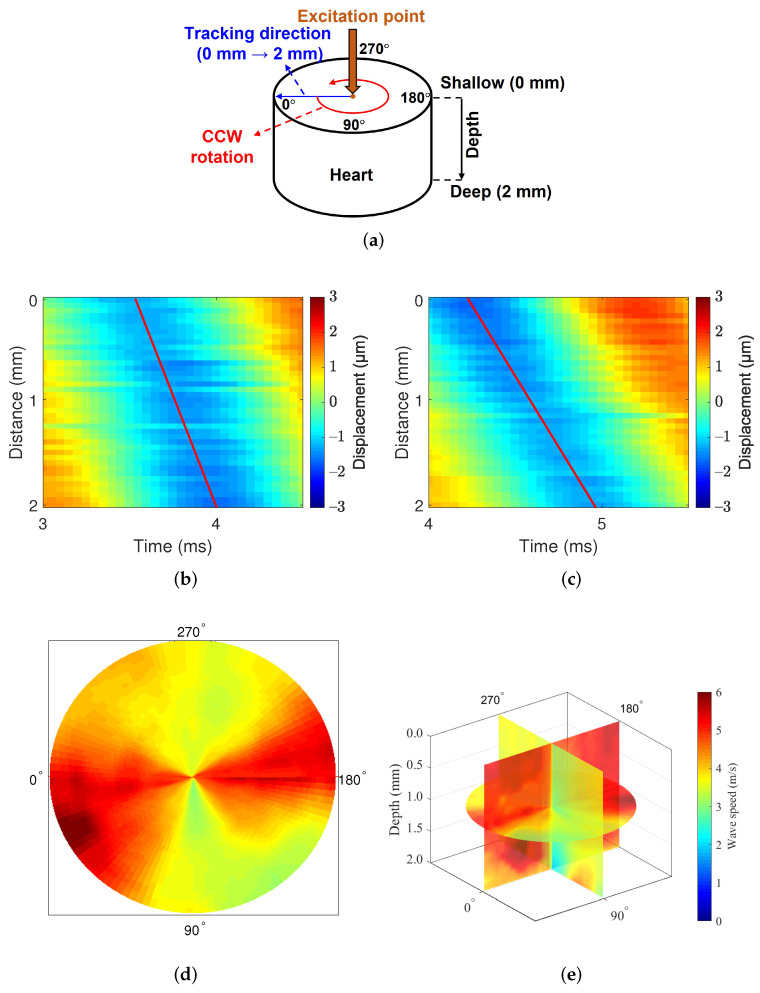
Heart image: directional dependence of the heart at the middle myocardium. The slope of the red line indicates the wave speed. (**a**) Data acquisition, (**b**) displacement map: circumferential direction, (**c**) displacement map: longitudinal direction, (**d**) heart image: 0 mm depth, and (**e**) heart image: 360${}^{\circ }$ (A depth of 0 mm does not mean the surface, but the point at which the ultrasound transducer started measuring inside of the tissue).

Heart experiments were designed to identify mechanical anisotropy. Figure 6a describes the experimental methods. The measurement area were middle-myocardium, and we measured the directional mechanics of the middle-myocardium by obtaining the wave speed at the depth of 2∼4 mm from the surface. The myocardium is started at a depth of 1∼2 mm from the heart wall, because the epicardial layer is thin around the middle area of the heart.

At each acquisition, the rotational stage rotated the heart tissue 360${}^{\circ }$ toward the counterclockwise direction at 3${}^{\circ }$ intervals, and data were acquired with 2 mm (depth) × 2 mm (width) at each angle. Figure 6b,c show the displacement maps of in the swine heart; slope of displacement maps represent the wave speed along the longitudinal and circumferential direction of the heart. The slope is steeper in the circumferential direction, which means wave speed along the circumferential direction is faster than along the longitudinal direction. Figure 6d shows the 360${}^{\circ }$ rotation image at the surface of the mid-epicardial tissue, and the measurement direction at 0${}^{\circ }$ represents the circumferential direction. The measured wave speed was relatively high around the angle 0${}^{\circ }$ and 180${}^{\circ }$ and relatively low around the angle 90${}^{\circ }$ and 270${}^{\circ }$. This result shows that the heart has anisotropic mechanical properties, consistent with results from other studies [19,28,29]. Figure 6e show the results of the rotation image with three dimensions, which validate the above results on both the tissue surface and inside.

The system was used to measure swine hearts in the longitudinal, circumferential, and radial directions. All measurements were performed at a depth of 0 mm. The circumferential and longitudinal directions represent the wave propagation directions at 0${}^{\circ }$ and 90${}^{\circ }$, respectively, in Figure 6a. The radial direction refers to the direction from the surface to the inside of the tissue. For this reason, tissues had to be placed vertically to detect the mechanical wave in the radial direction.

**Figure 7:**
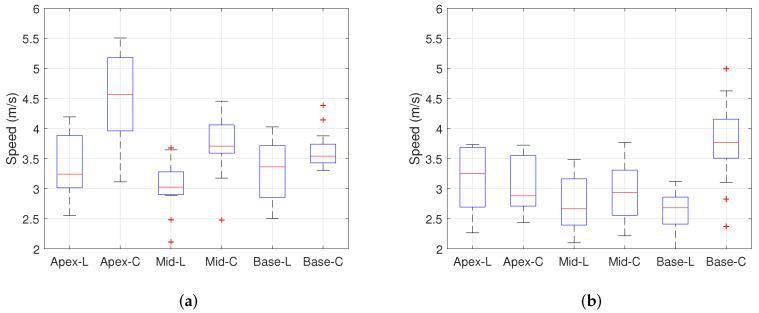

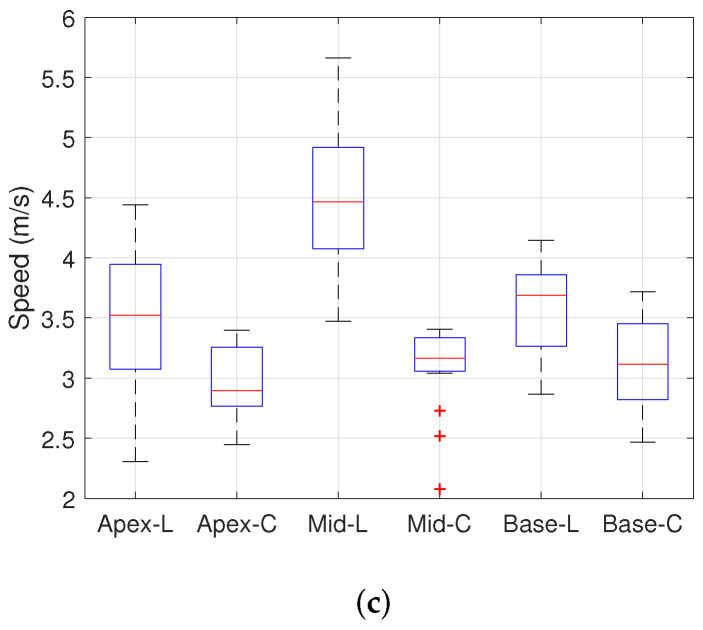
Boxplot for analyzing the wave speed depending on both location and layer. Red line: median, Upper black line: maximum, Lower black line: minimum, Top of the blue box: first quartile (25 %), Bottom of the blue box: third quartile (75 %), Red cross mark: outliers. (**a**) Epicardium, (**b**) Myocardium, and (**c**) Endocardium (L: longitudinal, C: circumferential).

Table 1 shows the wave speed values measured at the different layers and locations for 15 swine hearts. Each entry is the average of 45 wave speeds taken from 15 samples and repeated three times for each sample. For each entry, the values are given in the format of the mean ± standard deviation. A total of 21 data points are presented for the locations, layers, and fixed measurement directions. The wave speed in the radial direction at both the epicardium and endocardium was not measured because the radial widths in both layers were too thin. In the epicardium, the wave speed was faster in the circumferential direction than in the longitudinal direction. By contrast, the wave speed was faster in the longitudinal direction than in the circumferential direction in the endocardium. These results are visibly shown in Figure 7. In addition, the wave speed in the circumferential and longitudinal direction were significantly different at the Apex, Mid, and Base. In summary, the wave speed differs significantly depending on the measurement layer and location when the measurement direction is fixed. Table 2 shows the statistical meaning of the results in Table 1. This table provides the *p*-value of the independent two-sample *t*-test between the wave speed for the different directions in each layer and each location. In the table, the *p*-values for the myocardium were corrected by Bonferroni correction, because the comparisons were performed three times among three groups. Each wave speed is significantly different (*p* < 0.05) from others except for the case of the myocardium and base free wall.

#### 3.2.2. Cartilage Experiments

**Figure 8:**
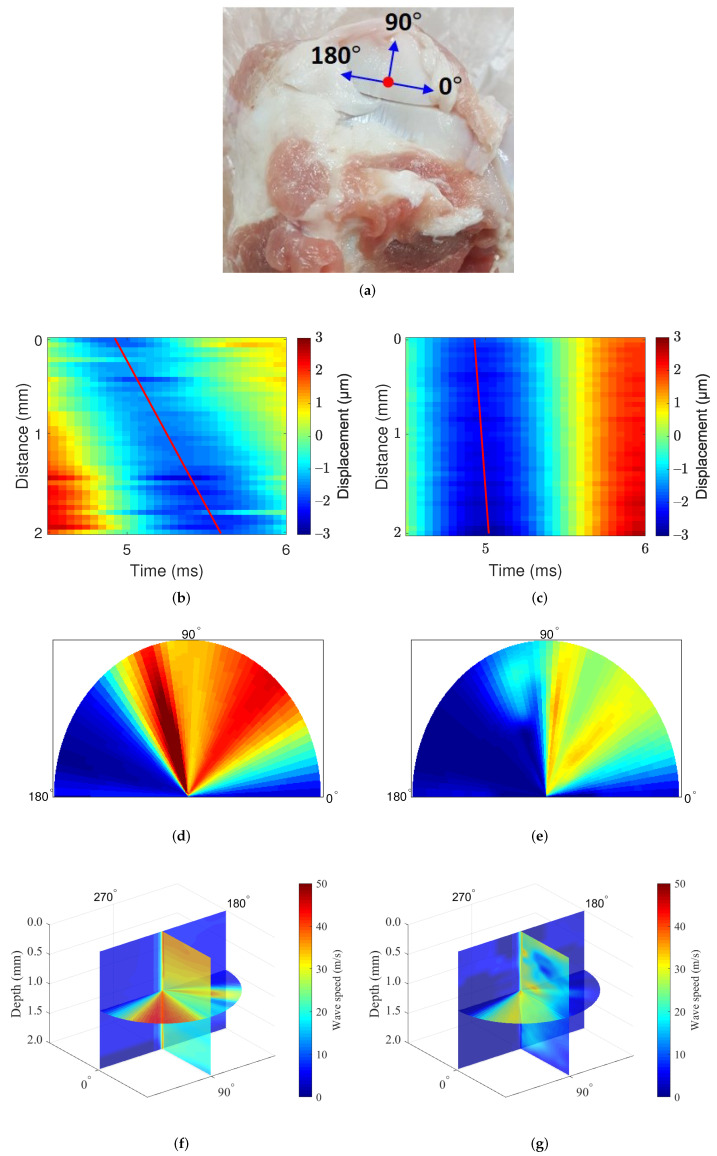
Cartilage image: directional dependence of the cartilage. The slope of the red line indicates the wave speed. (**a**) Cartilage image, (**b**) displacement map: circumferential direction, (**c**) displacement map: longitudinal direction, (**d**) cartilage image1: 0 mm depth, (**e**) cartilage image2: 0 mm depth, (**f**) cartilage image1: 180${}^{\circ }$, and (**g**) cartilage image2: 180${}^{\circ }$ (A depth of 0 mm does not mean the surface, but the point at which the ultrasound transducer started measuring inside of the tissue).

This experiment was designed to identify the directional mechanics of the cartilage tissues with our mechanical wave imaging system. Figure 8d represents the cartilage of the swine forelimb as the area measured with the imaging system. As the cartilage was too small to acquire 360${}^{\circ }$ image, data were acquired at the angle from 0${}^{\circ }$ to 180${}^{\circ }$. Figure 8e,f show the displacement maps of in the cartilage, slope of displacement maps represent the wave speed along the longitudinal and circumferential direction of the cartilage. The slope is highly steep in circumferential direction, which means wave speed along the circumferential direction is much faster than along the longitudinal direction. Figure 8g–j show the results from this experiment. The wave speed was measured in two different cartilage samples, and the measurement speed ranged from approximately 5 m/s to approximately 50 m/s. The obtained wave speed was slow at angles of approximately 0${}^{\circ }$ and 180${}^{\circ }$ and fast at angles of 10∼110${}^{\circ }$. Although the speed obtained from the two different cartilage samples differed, these results clearly show that cartilage has anisotropic mechanical properties. In addition, these results show that the directional mechanics of relatively hard tissue such as cartilage can be observed with our imaging system.

## 4. Discussion

We incorporated a mechanical shaker and motorized rotation stage into a wave elastography system for the diagnostics of mechanically anisotropic tissues with a wide range of stiffnesses. Two types of experiments (heart and cartilage) were designed to validate the feasibility of our imaging system on actual tissues.

Swine heart experiments were performed on 15 pigs, the results of which are given in in Table 1. First we note that the measured wave speed was mainly distributed from 2.5 m/s to 5.5 m/s, while, in other studies [30,31], the measured wave speed ranges from approximately 1 m/s to 5 m/s. This comparison supports that the proposed system is well calibrated.

We clearly observed that the wave speeds differ depending on the measurement direction, location, and layer. The differences are statistically significant as shown by the *p*-values in Table 2 in most cases except for the myocardium and base free wall. These results indicate that the differences in the wave speed between the longitudinal and circumferential directions in the epicardium and endocardium are statistically significant. Specifically, in the epicardium, the wave speed is faster in the circumferential direction than in the longitudinal direction, and in the endocardium, the speed is faster in the longitudinal direction than in the circumferential direction. In the myocardium, however, the differences in the measured wave speed according to measurement direction are not distinguishable. These results indicate that the directional mechanics differ by the measurement layers, which is consistent with the results from other studies [19,28,29]. We also observe that the directional mechanics differ by the measurement locations (apex, mid, and base) as well. As shown in Figure 7, the difference between the longitudinal direction and circumferential direction varied for different measurement locations. This means that the fiber orientation depends not only on the layer but also on the location of the tissue. Thus, acquiring data along a fixed direction may result in misdiagnostics of the pathological state of tissue that is mechanically anisotropic.

Figure 6 shows the directional dependence of mechanical wave imaging with high angular resolution. Clearly, the measured speeds change even over a small degree of rotation, which supports the necessity of high angular resolution. The proposed system is able to measure the wave speed with high angular resolution to reduce bias caused by misalignment of the measurement direction.

Previous studies developed the mechanical wave imaging system to measure directional mechanics [19,28]. In these studies, the imaging system manually rotate tissues or ultrasound probes. However, the manual rotation make the imaging process time consuming and less accurate. The proposed imaging system ensures accurate and fast data acquisition due to the usage of the motorized rotational stage. Furthermore, previous studies do not provided the high spatial resolution because these imaging systems use the commercial ultrasound probes which do not have enough center frequency. However, high-frequency ultrasound imaging in mechanical wave elastography would be beneficial for the better diagnostics of the diseases as shown in the previous study [3]. Thus, high-frequency ultrasound elastrography is required. The proposed imaging system uses the ultrasound transducer that has center frequency of 38 MHz, and the spatial resolution is were determined to be 144 μm and 168 μm, respectively. The above advantages make diagnostics reliable. These results indicate the strength of our study, which are not described in the previous studies.

We note that it is possible to identify the direction of the fiber orientation using the proposed system: it is the direction in which the mechanical wave propagates at the fastest. This is due to the fact that wave speed is fastest along the direction of the fiber orientation in anisotropic materials [28]. Identifying the fiber orientation would not have been possible, or at least required additional processing, if the angular resolution of the proposed system were not high enough. In the future, the identified fiber orientation may be compared with other methods such as diffusion tensor imaging, elastic tensor imaging, or backscatter tensor imaging that estimate the fiber orientation [32,33].

Strain-based elastography can also measure direction-dependent mechanical properties by measuring the mechanical properties in multiple directions. In order for this, the tissue sample must be rotated in three-dimensional manner in the measurement device. Incorporating the automated rotation in three-dimensional manner for repeatable and accurate measurement may require a complex mechanism. Most importantly, strain-based elastography cannot quantitatively measure the mechanical properties due to the absence of a stress measuring sensor. Thus, the direction-dependent mechanical properties are not quantitatively distinguished in the strain-based elastography. Based on the two points discussed, we view that the proposed imaging system is superior to strain-based elastography and its potential extensions.

Next we discuss the measurement of directional mechanics in swine cartilage using mechanical wave imaging, which was performed for the first time to the best of our knowledge. Figure 8 shows the directional mechanics of the swine cartilage obtained by our imaging system. We first notice that the measured speeds exhibit a large discrepancy between the two samples. Note that the two samples differ in size and shape, and they came from two different pigs. Explanations are in order. We raise the possibility of the proposed imaging system not acquiring quantitatively accurate measurement values for the cartilage. We suspect that the inaccuracy may be attributed to the cartilage sample thickness of 2 mm. The wave in the sample may behave as a guided wave which exhibits dispersion. In this case, the exact mechanical properties of cartilage may not be directly calculated by the propagation speed of a wave measured at a single frequency. Using wave propagation speeds at various frequencies, a dispersion analysis may be necessary in order to correctly measure the mechanical properties for such a thin sample. Furthermore, a rigorous ex vivo experiment with a large number of samples may be necessary.

Nevertheless, the results demonstrate that the proposed imaging system can measure the wave propagation speed of relatively hard tissues such as cartilage and that a mechanical wave elastography method can measure the directional mechanics of cartilage qualitatively. In addition, the observed mechanical properties in cartilage are consistent with the measured direction-dependence and the stiffness greater than thousands of kPa in the previous studies [34,35]. Although we did not perform dispersion analysis in this study, qualitative measurement of directional mechanical properties in cartilage could be realized by using the proposed system, thus demonstrating that the proposed imaging system can be used to analyze the directional mechanics of tissues with a wide range of stiffnesses. By applying the dispersion analysis method to the proposed system, a better quantitative measurement of directional mechanical properties in cartilage can be achieved. The related work remains as a future study.

While this system is useful for ex vivo evaluation, this system cannot be used for in vivo evaluation due to its size (bulkiness) and low penetration depth. Consequently, the use of this system is limited to ex-vivo research, and modifications enabling a future clinical set-up are required. Ultrasound imaging has been used to image structure of heart and cartilage. Therefore, in order to apply this system in clinical tests, the development of a method for generating mechanical waves inside the human body is necessary. There are several methods to achieve such; first, a compact vibrator can be used for mechanical wave source. The compact vibrator should be inserted in intracardiac probe. The vibrator mounted on probe contacts the cardiac tissue and directly displaces the cardiac tissue to generate a mechanical wave. Alternatively, the displacement may take place in tissue by generating large mechanical wave at the skin near the cartilage or heart. In such systems, the mechanical shaker and ultrasound probe can be physically separated. Clinical examination might be performed after these developments of mechanical wave source. The rotation stage is another problem to be solved in order to apply the imaging system inside the human body. At least three ultrasound transducers should be mounted on the catheter, each of which detects the displacement in a different measurement direction. The wave speed according to fiber orientation and the wave speed perpendicular to fiber orientation can be estimated by applying the measured wave speed to an anisotropic material model. By solving these problems, this imaging system could be applying into clinical test.

## 5. Conclusions

In this study, we designed a high-frequency (38 MHz) mechanical wave imaging system with a mechanical vibrator capable of measuring the directional mechanics of tissues. The developed system was proposed for ex vivo evaluation of the directional mechanics of tissue at various angles. The imaging system was validated in experiments with phantoms and actual swine heart and cartilage. Based on the phantom experiments, the axial and lateral resolutions were calculated as 168 μm and 144 μm, respectively. The swine heart experiments demonstrated that the directional mechanics of tissue differ according to the measurement location due to variations in fiber orientation with measurement location. In addition, imaging of the heart (360${}^{\circ }$) clearly showed where the wave speed was high (fiber direction) and low (perpendicular to the fiber), which has implications for facilitating tissue diagnostics. In addition to the heart experiments, experiments were performed for the first time with swine cartilage tissue. The results showed that the proposed system is feasible for measuring the directional mechanics of tissues even if the tissue is very hard. In summary, the proposed system is able to measure the directional mechanics of tissues ranging from soft to hard with high angular resolution, which is helpful for diagnosing tissues with mechanically anisotropic properties.

## Figures and Tables

**Table 1:** Experimental results: wave speed in swine hearts.

Layer	Direction	Location		
		Apex	Mid	Base
Epicardium	Longitudinal	3.42±0.51	3.01±0.51	3.33±0.50
	Circumferential	4.55±0.70	3.75±0.50	3.64±0.30
Endocardium	Longitudinal	3.54±0.60	4.52±0.63	3.60±0.40
	Circumferential	2.97±0.27	3.08±0.37	3.13±0.38
Middle-myocardium	Longitudinal	3.11±0.55	2.75±0.43	2.64±0.33
	Circumferential	3.05±0.45	2.93±0.46	3.75±0.67
	Radial	2.82±0.40	2.70±0.40	2.85±0.45

Unit: m/s.

**Table 2:** *p*-values for the locations. (**a**) Apex, (**b**) Mid, and (**c**) Base. (L: longitudinal, C: circumferential, and R: radial).

	Epi-C	Endo-C	Myo-C	Myo-R
(**a**)				
Epi-L	*p* = 0.008			
Endo-L		*p* = 0.014		
Myo-L			*p* = 1.000	*p* = 0.288
Myo-R			*p* = 0.435	
(**b**)				
Epi-L	*p* = 0.002			
Endo-L		*p* ≈ 0		
Myo-L			*p* = 1.000	*p* = 0.183
Myo-R			*p* = 1.000	
(**c**)				
Epi-L	*p* = 0.123			
Endo-L		*p* = 0.005		
Myo-L			*p* ≈ 0	*p* = 1.000
Myo-R			*p* = 0.003	

## Data Availability

The data presented in this study may be available upon request to the corresponding author.
